# ACUPUNCTURE AND ACUPRESSURE FOR DEMENTIA BEHAVIORAL AND PSYCHOLOGICAL SYMPTOMS: A SCOPING REVIEW

**DOI:** 10.1093/geroni/igz038.427

**Published:** 2019-11-08

**Authors:** Melissa L Harris, Laura M Struble, Marita Titler

**Affiliations:** University of Michigan, Ann Arbor, Michigan, United States

## Abstract

This scoping review synthesized the literature on acupuncture and acupressure therapy for treating behavioral and psychological symptoms of dementia (BPSD). BPSD affect most people with dementia and are overwhelming for caregivers and healthcare providers. Current evidence encourages use of nonpharmacologic interventions for BPSD due to the dangers of psychotropic medications. Acupuncture and acupressure present as alternative treatment options for BPSD. Systematic reviews examined acupuncture therapy for improving cognitive function, but not acupuncture and/or acupressure for BPSD. The methodology outlined by Joanna Briggs Institute Reviewers’ Manual guided this scoping review. Databases searched were PubMed, CINAHL, Embase, PsychINFO and AgeLine. Study inclusion criteria were published in English, subjects with dementia, acupuncture or acupressure tested and outcome measures included at least one BPSD. Gray literature was excluded. 836 citations were screened by title and abstract. 57 full texts were reviewed to determine inclusion criteria and 15 studies were retained. Nine studies examined acupressure and 6 examined acupuncture. Eight studies were RCT, 11 were conducted in China and 10 were conducted in long-term care. The percent of studies with statistically significant improvements in BPSD outcomes measured were: ADL’s (75%), agitation (100%), anxiety (67%), depression (100%), mood (100%), neuropsychological disturbances (67%) and sleep disturbances (100%). The therapies were safe and participant satisfaction was high across studies. Variations in research designs, outcome measures and incomplete descriptions of acupoints limit interpretations about effectiveness of these interventions for BPSD. Additional RCTs are needed to improve generalizability and to evaluate dosage/methods of acupressure and acupuncture for BPSD.

